# Effectiveness of Dual-Focus Magnification on Confidence Levels in Optical Diagnosis of Small Colorectal Polyps

**DOI:** 10.7759/cureus.53545

**Published:** 2024-02-04

**Authors:** Tien M Huynh, Quang D Le, Nhan Q Le, Huy M Le, Duc T Quach

**Affiliations:** 1 Department of Internal Medicine, University of Medicine and Pharmacy at Ho Chi Minh City, Ho Chi Minh City, VNM; 2 Department of GI Endoscopy, University Medical Center Ho Chi Minh City, Ho Chi Minh City, VNM; 3 Department of Endoscopy, Nhan dan Gia Dinh Hospital, Ho Chi Minh City, VNM; 4 Department of Surgical Pathology, University of Medicine and Pharmacy at Ho Chi Minh City, Ho Chi Minh City, VNM

**Keywords:** vietnam, confidence level, optical diagnosis, dual-focus, narrow banding imaging, small colorectal polyp

## Abstract

Background and objectives

Achieving accurate real-time optical diagnoses of colorectal polyps with high-confidence predictions is crucial for appropriate decision-making in daily practice. The dual-focus (DF) magnification mode helps endoscopists scrutinize subtle features of polyp surfaces and vessel patterns. This prospective study aimed to evaluate the impact of DF imaging on enhancing the rate of high-confidence narrow-band imaging (NBI)-based optical diagnosis.

Methods

Consecutive adult patients who underwent colonoscopy and had small colorectal polyps (<10 mm) were enrolled between September 2022 and May 2023. The optical diagnosis of each polyp was evaluated during colonoscopy in two stages by the same endoscopist, utilizing NBI with DF magnification (NDB-DF). A confidence level was assigned to each prediction. High confidence was indicated by clinical judgment when a polyp exhibited distinctive features associated solely with one histological subtype and lacked characteristics of any other subtype. All procedures were carried out with a prototype 190 series Exera III NBI system (Olympus Corporation, Tokyo, Japan) with DF magnification.

Results

The study included 413 patients with 623 polyps, comprising 483 ≤ 5 mm and 140 measuring 6-9 mm. The majority were low-grade adenomas (343 lesions), with 17 identified as high-grade adenomas, and none characterized as deep submucosal invasive carcinomas. NBI-DF significantly improved the rate of high-confidence optical diagnoses compared to NBI for both ≤ 5 mm polyps (93.1% vs. 87.5%, p < 0.0001) and 6-9 mm polyps (97.9% vs. 94.2%, p = 0.03). Furthermore, DF significantly facilitated the assessment of microvessel and surface pattern criteria (p < 0.01).

Conclusion

DF magnification markedly enhanced the rate of high-confidence NBI-based optical predictions for small colorectal polyps. This technique demonstrates the potential for improving the diagnostic yield in real-time optical diagnosis of colorectal polyps in the Vietnamese setting.

## Introduction

Advanced imaging technology facilitates real-time assessment of polyp histology during colonoscopy, enhancing optical endoscopic diagnosis. Among the various endoscopic image enhancement methods, narrow-band imaging (NBI) has emerged as a commonly applied tool since its introduction in 2006 [1]. Its widespread use in general clinical practice for diagnosing colorectal lesions attests to its popularity.

Several studies affirm the safety and feasibility of optical diagnosis for small colorectal polyps, highlighting its comparability to histology [2-6]. Confidence in the assessment of endoscopists plays a pivotal role in optical diagnosis. Only lesions in which the endoscopist has a high confidence level should be considered for optical diagnosis. The success of the optical diagnosis strategy in clinical practice hinges on the calibration and standardization achievable through confidence levels, accommodating variations in diagnostic abilities among endoscopists [7]. Previous reports underscore that high-confidence optical diagnosis via NBI allows expert endoscopists to achieve superior diagnostic performance [5,6,8]. Increasing the rates of high-confidence NBI-based optical diagnosis holds the potential to enhance real-time diagnostic capabilities in vivo, fostering the widespread adoption of this technique in routine clinical practice.

Currently, the reported rates of high-confidence NBI-based optical diagnosis during real-time colonoscopy without magnification range from 75% to 80% [1]. The availability of optical zoom endoscopy, which improves the accuracy and rate of high-confidence optical diagnosis with NBI, is notably limited to regions outside Japan. A promising alternative to optical zoom is the dual-focus (DF) mode, a technique capable of providing comparable images through a simple button push [9]. Building on our prior studies demonstrating the high diagnostic performance of NBI-DF for small colorectal polyps [10], the current study aimed to evaluate whether DF magnification could enhance the rate of high-confidence NBI-based neoplastic optical diagnosis for small colorectal lesions.

## Materials and methods

Study design and participants

This cross-sectional, single-center observational study was conducted at the University Medical Center in Ho Chi Minh City, Vietnam, from October 2022 to May 2023. The inclusion criterion was consecutive adult patients (aged 18 or older) who underwent elective colonoscopy for screening, surveillance, or diagnostic workup, specifically those with small colorectal polyps (< 10 mm) requiring histologic examination post-surgery. The exclusion criteria included individuals who presented solely with colorectal polyps more than 10 mm in length or without endoscopically detected polyps, who had poor bowel preparation, who had a history of inflammatory bowel disease, colectomy, colon cancer, or polyposis syndrome, who used antiplatelet or anticoagulant drugs to prevent polyp removal, who underwent polypectomy but could not retrieve the polyp for histopathological analysis, and who were unable to provide informed consent.

Endoscopy prediction procedure

All procedures were performed utilizing a prototype 190 series EVIS EXERA III CV-190 system (Olympus Corporation, Tokyo, Japan) featuring DF capability, coupled with colonoscopes (CF-HQ190I). A transparent cap was employed to optimize the focus. NBI and DF were seamlessly incorporated as easily interchangeable push-button techniques alongside traditional white light and standard focus.

Two highly experienced endoscopists, NQL and QDL, who specialized in colonoscopy with extensive proficiency in NBI (> 1000 cases), conducted the procedures. They received training to be proficient in using NBI and were familiar with the NBI International Colorectal Endoscopy (NICE) classification. All polyps identified under white light imaging during colonoscopy underwent rigorous washing and examination in two phases: first with NBI and subsequently with NBI-DF. The recorded details included polyp location, size estimated with biopsy forceps or polypectomy snare, and shape classified according to the Paris classification [11].

Endoscopists employed the NICE classification (NICE 1, nonneoplastic lesion; NICE 2, adenoma; NICE 3, deep submucosal invasive carcinoma) to categorize polyp types based on color, vessel, and surface pattern [1]. They also expressed their confidence levels (high or low) in the predictive assessments, with "high confidence" denoted clinical judgment when a polyp displayed distinct features associated with one histological subtype and lacked features of any other subtype [7,10]. Any uncertainty or doubt prompted a low-confidence prediction. An independent observer (TMH) documented the diagnosis at each stage, preventing alterations to predictions in subsequent steps.

Histopathological investigations

All polyps underwent resection or biopsy to undergo histopathological evaluation, serving as the reference standard. A proficient gastrointestinal pathologist (HML), who was unaware of the endoscopic diagnosis, systematically categorized all specimens in accordance with the World Health Organization classification [12]. Lesions identified histopathologically as adenomas, sessile serrated adenomas, traditional serrated adenomas, or carcinomas were categorized as neoplastic lesions. Conversely, those including hyperplastic and inflammatory polyps were classified as non-neoplastic lesions.

Determining sample size and analyzing statistics

In our study, we determined the sample size for comparing two related proportions using the McNemar test with the formula: n=$(\frac{(p1+p2)}{p1(1-p2)+p2(1-p1)}x \frac{1}{power}x\frac{1}{significance level})$, where p1​ and p2​ represent high-confidence rates of 90% (NBI-DF) and 80% (NBI), respectively. Employing a two-sided 5% significance level with 80% power, a sample size of 313 consecutive polyps was required [3,8]. The predicted and final polyp histology results were evaluated to determine the sensitivity, specificity, accuracy, positive predictive value (PPV), and negative predictive value (NPV) of NBI and NBI+NBI-DF. Additionally, a secondary analysis was carried out using the chi-square test. A two-sided p-value less than 0.05 was considered to indicate statistical significance. The statistical analysis was performed using IBM SPSS Statistics for Windows, Version 23.0 (Released 2015; IBM Corp., Armonk, New York, United States).

Ethical consideration

The study, conducted in accordance with the Declaration of Helsinki 1975, received ethical approval from the Board of Ethics in Biomedical Research at the University of Medicine and Pharmacy at Ho Chi Minh City, Vietnam (approval number: 146/HĐĐĐ-ĐHYD, signed in September 2022). All participating patients provided written informed consent before undergoing examinations.

## Results

Clinicopathologic features

**Figure 1:**
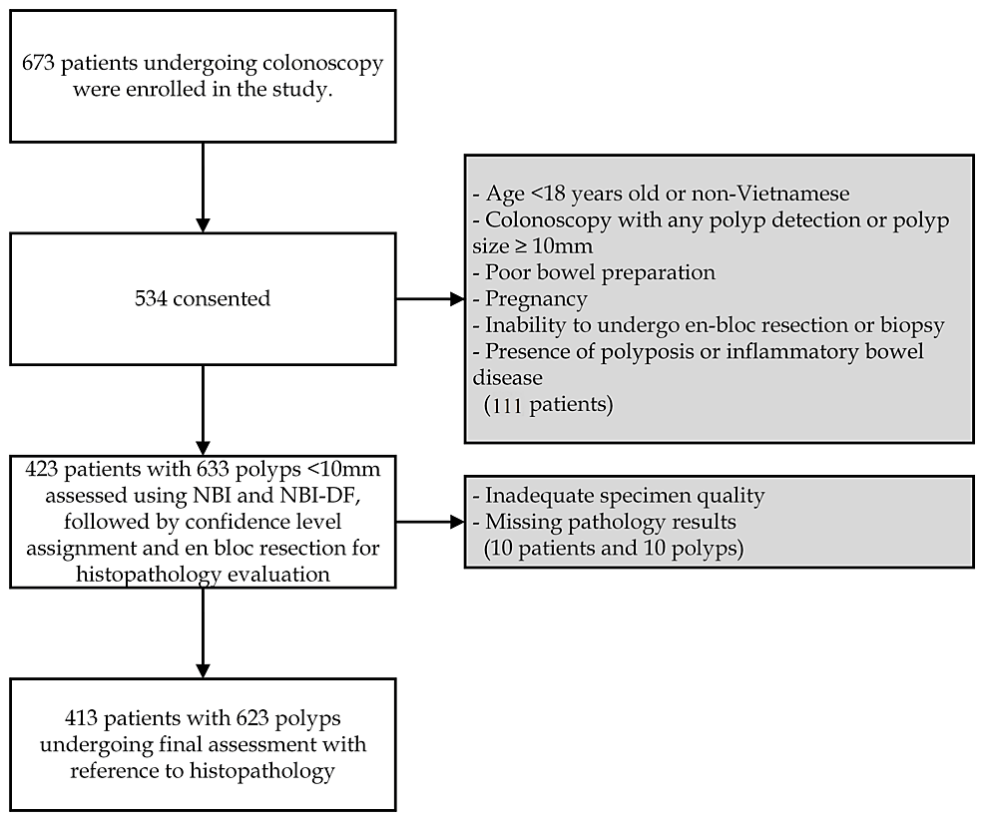
Flowchart of the study

Out of the 673 patients invited to participate, a prospective analysis was conducted on 413 patients, encompassing 623 polyps measuring less than 10 mm (Figure 1). Table 1 details the characteristics of the eligible patients and the resected polyps. Among the 623 polyps, 483 were classified in the group with a size of ≤ 5 mm in length, while 140 were categorized in the group with a size of 6-9 mm. According to the Paris classification, the macroscopic shapes included 267 protruded lesions (0-Is, Ip) and 356 flat lesions (0-IIa). The most prevalent histological finding was tubular adenoma, accounting for 343 out of the 623 lesions (54.1%). Twenty-four lesions (3.8% of the total 623) were classified as advanced. These included 10 low-grade villotubular adenomas, two high-grade villotubular adenomas, one villous adenoma, six high-grade tubular adenomas, and five traditional serrated adenomas. However, due to the exclusive focus on polyps smaller than 10 mm, the study did not calculate the polyp detection rate or adenoma detection rate.

**Table 1:** Participant (N=413) and resected polyp (N=623) characteristics Data presented as n (%), unless otherwise indicated.

Characteristic		Findings, n (%)
Patient characteristics (N=413)	Gender	
	Male	237 (57.3)
	Female	176 (42,7)
	Age (years), mean ± SD	59.4 ± 13.5
	Indication for colonoscopy	
	Screening	129 (31.2)
	Surveillance	49 (11.8)
	Symptoms	235 (57.0)
Endoscopic findings (N=623)	Location	
	Cecum	35 (5.6)
	Ascending	91 (14.6)
	Transverse	124 (19.9)
	Descending	82 (13.2)
	Sigmoid	191 (30.6)
	Rectum	100 (16.1)
	Mean size (mm), mean ± SD,	3.8 ± 2.2
	Lesion morphology	
	Type 0-Ila	356 (57.1)
	Type 0-Is	251 (40.3)
	Type 0-Ip	16 (2.6)
	Polyp resection	
	Cold biopsy	436 (70)
	Cold snare	129 (20.7)
	Hot snare	58 (9.3)
Histological findings (N=623)	Non-neoplastic	
	Hyperplastic	111 (17.8)
	Other non-hyperplastic (inflammatory polyps, Lymphoid polyp)	146 (23.4)
	Neoplastic	
	Tubular adenoma	343 (55.1)
	Tubulovillous adenoma	12 (1.7)
	Villous adenoma	1 (0.1)
	Traditional serrated adenoma	5 (0.8)
	Sessile serrated lesion	7 (1.1)

Rates of high-confidence optical diagnosis

**Figure 2:**
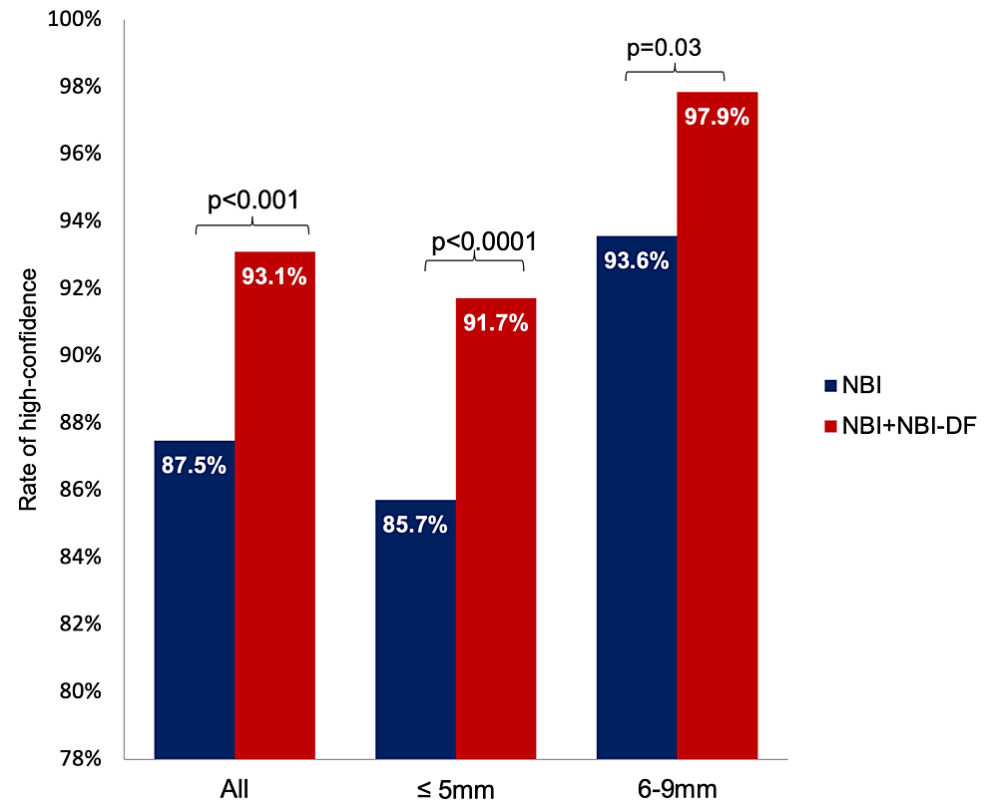
Rates of high-confidence optical diagnosis with NBI and NBI+NBI-DF stratified by size NBI: narrow banding imaging; NBI-DF: narrow banding imaging with dual focus p: McNemar test

Figure 2 and Table 2 depict the rates of high-confidence optical diagnosis using NBI and NBI+NBI-DF. A notably greater percentage of the 623 polyps were predicted with high confidence by NBI+NBI-DF (87.5%, n=580) than by NBI alone (93.1%, n=545; p<0.001). After stratifying the polyps by size, the DF mode significantly increased the rate of high-confidence optical diagnosis in both the ≤5 mm group (p<0.0001) and the 6-9 mm group (p=0.03).

**Table 2:** Effect of the dual focus magnification on the level of confidence and diagnostic performance of narrow banding imaging NBI: narrow banding imaging; NBI-DF: narrow banding imaging with dual focus; HC: high confidence; LC: low confidence

	NBI	Accuracy (%)	Sensitivity (%)	NBI+NBI-DF	Accuracy (%)	Sensitivity (%)
All (n=623)	HC (n=545)	86.3	87.0	HC (n=545)	89.5	93.7
				LC (n=0)	-	-
	LC (n=78)	51.6	51.6	HC (n=42)	76.2	76.5
				Low (n=36)	45.3	49.5
≤ 5 mm (n=483)	HC (n=414)	87.5	82.8	HC (n=414)	91.2	91.1
				LC (n=0)	-	
	LC (n=69)	81.2	82.1	HC (n=36)	83.3	80.0
				LC (n=33)	63.6	69.2
6-9 mm (n=140)	HC (n=131)	82.4	95.1	HC (n=131)	86.3	100
				LC (n=0)	-	-
	LC (n=9)	50.0	50.0	HC (n=6)	66.7	50.0
				LC (n=3)	33.3	50.0

Table 2 demonstrates the impact of DF magnification on confidence levels concerning accuracy and sensitivity in NBI, categorized by polyp size. Within the subset of 69 polyps ≤ 5 mm in size, which were initially predicted to have low confidence by NBI (with an accuracy of 81.2% and sensitivity of 82.1%), 36 (52.7%) polyps were subsequently classified with high confidence by NBI+NBI-DF. This transition led to an enhanced accuracy of 83.3% and a sensitivity of 80.0%. Similarly, among the nine polyps in the size range of 6-9 mm that were initially predicted with low confidence (with an accuracy of 50.0% and sensitivity of 50%) by NBI, six (66.7%) were subsequently predicted with high confidence by NBI+NBI-DF. This shift increased the accuracy by 66.7% while maintaining the same sensitivity of 50%. Conversely, when lesions received low-confidence predictions in both NBI and NBI-DF, there was a decrease in sensitivity and accuracy (from 51.6% to 45.3% in accuracy and from 51.6% to 49.5% in sensitivity). However, it is worth noting that these improvements in accuracy and sensitivity with NBI+NBI-DF did not significantly differ between the ≤ 5 mm group (p = 0.27) and the 6-9 mm group (p = 0.43).

Performance characteristics of optical diagnosis with NBI and confidence level

The performance characteristics of optical diagnosis with NBI, presented with varying levels of confidence, are detailed in Tables 4, 5. Overall, the incorporation of the DF mode demonstrated a notable improvement in the accuracy and sensitivity of neoplastic prediction (82.1% vs. 87.0% and 80.1% vs. 90.1%, p<0.001), particularly for lesions smaller than 5 mm (p<0.001); moreover, no significant improvement was observed in the 6-9 mm size group (p=0.12). Additionally, there were no discernible differences in performance characteristics between NBI-DF and NBI in the high-confidence prediction group (p=0.23). In the NBI+NBI-DF mode, polyps 6-9 mm in length were predicted with high confidence and exhibited higher sensitivity but lower accuracy than those ≤5 mm in terms of neoplastic prediction (98.1% vs 89.3% in sensitivity and 90.3% vs 84.0% in accuracy).

**Table 3:** Performance characteristics of NBI and NBI+NBI-DF with levels of confidence NBI: narrowband imaging; NBI-DF: narrow-band imaging with dual focus; PPV: positive predictive value; PPV: negative predictive value Data presented as percentage (95%CI).

Diagnostic performance	Confidence level	All, % (95%CI)		≤ 5 mm, % (95%CI)		6 – 9 mm, % (95%CI)	
		NBI	NBI +NBI-DF	NBI	NBI +NBI-DF	NBI	NBI +NBI-DF, % (95%CI)
Accuracy	High	86.3 (83.1-89.0)	88.8 (85.9-91.3)	87.5 (83.9-90.5)	90.3 (87.2-93.0)	82.4 (74.8-88.5)	84.0 (76.7-89.7)
	Low	51.6 (38.6-64.5)	65.1 (49.1-79.0)	81.2 (69.9-89.6)	65.0 (48.3-79.4)	50.0 (26.0-74.0)	-
	All	82.1 (78.9-85.10	87.2 (84.3-89.7)	83.02 (79.4-86.3)	88.2 (85.0-91.0)	78.5 (71.1-84.8)	83.5 ( 76.2-89.2)
Sensitivity	High	87.0 (82.7-90.6)	92.1 (88.7-94.8)	82.8 (76.8-87.8)	89.3 (84.5-93.1)	95.1 (88.9-98.4)	98.1 (93.4-99.8)
	Low	51.6 (38.6-64.5)	68.8 (50-83.9)	82.1 (66.5-92.5)	70.0 (50.6-85.3)	50.0 (21.1-78.9)	50 (1.26-98.7)
	All	81.0 (76.6-84.9)	90.1 (86.5-92.9)	77.2 (71.5-82.2)	87.7 (83.0-91.5)	90.4 ( 83.4-95.1)	97.2 ( 92.1-99.4)
Specificity	High	85.3 (80.2-89.5)	84.4 (79.3-88.7)	91.7 (76.8-87.8)	91.3 (86.8-94.7)	37.9 (20.7-57.8)	35.5 (19.2-54.6)
	Low	62.5 (35.4-84.8)	54.6 (23.4-83.3)	80 (61.5-92.3)	50 (18.7-81.3)	50 (11.8-88.2)	-
	All	83.9 (78.9-88.2)	83.1 (78.1-87.5)	89.5 (84.8-93.2)	89.5 (84.8-93.2)	40.0 (28.9-57.9)	35.5 (19.2-54.6)
PPV	High	87.9 (84.2-90.8)	88.6 (85.4-91.2)	90.1 (85.4-93.4)	91.3 (87.2-94.2)	84.4 (80.2-87.8)	83.9 (80.0-87.1)
	Low	84.2 (73.1-91.3)	81.5 (68.9-89.8)	84.2 ( 72.0-91.8)	80.8 (68.4-89.1)	66.7 (42.9-84.2)	100 (2.5-100)
	All	87.5 (84.1-90.3)	88.1 (85.0-90.7)	89.1 (84.8-92.3)	90.2 (86.3-93.1)	83.1 (78.8-86.6)	84.0 (80.2-87.2)
NPV	High	84.3 (79.9-87.8)	89.1 (84.8-92.2)	85.3 (81.1-88.8)	89.3 (95.1-92.4)	68.8 (45.4-85.3)	84.6 (56.3-95.9)
	Low	25.0 (17.41-34.5)	37.5 (22.2-55.8)	77.4 (63.1-87.3)	35.7 (19.6-55.9)	33.3 (15.8-57.1)	-
	All	76.1 (71.8-79.8)	85.8 (81.5-89.2)	78.0 (73.7-81.7)	86.7 (82.6-90.2)	56.0 (38.9-71.8)	78.6 (52.2-92.5)

**Table 4:** Associations between real-time optical diagnoses and histological findings NBI: narrow banding imaging; NBI-DF: narrow banding imaging with dual focus; NICE: NBI International Colorectal Endoscopy

Optical diagnostic mode	NICE classification	Size	Confidence level	Final histology, n	
				Neoplastic	Non-neoplastic
NBI	NICE 1	All	High	39	209
			Low	30	10
		<5 mm	High	34	198
			Low	24	7
		6-9 mm	High	5	11
			Low	6	3
	NICE 2	All	High	261	36
			Low	32	6
		<5 mm	High	164	18
			Low	32	6
		6-9 mm	High	97	18
			Low	6	3
NBI+ NBI-DF	NICE 1	All	High	26	211
			Low	10	6
		<5 mm	High	24	200
			Low	9	5
		6-9 mm	High	2	11
			Low	1	0
	NICE 2	All	High	304	39
			Low	22	5
		<5 mm	High	200	19
			Low	21	5
		6-9 mm	High	104	20
			Low	1	0

Analysis of low-confidence lesions

When examining lesions with low confidence (Table 5), it was observed that the size of the polyps was significantly smaller than that of the high confidence (3.4 ± 2.1 mm vs. 3.9 ± 2.2 mm, p=0.03). These lesions were more commonly located on the right side (55.2%) and most exhibited 0-IIa morphology (65.4%). However, no significant differences were reported between patients with high and low confidence regarding location or morphology.

**Table 5:** Comparison of characteristics between high-confidence and low-confidence lesions NBI: narrow banding imaging; NBI-DF: narrow banding imaging with dual focus p: Chi-square test Data presented as n (%), unless otherwise indicated.

Characteristic		Confidence level		p-value
		High, n (%)	Low, n (%)	
Lesion (N=623)		545 (87.5)	78 (12.5)	
Size (mm), mean±SD		3.9±2.2	3.4±2.1	0.03
Location distribution	Right side (cecum, ascending colon, transverse colon)	219 (40.2)	43 (55.1)	0.06
	Left side (descending colon, sigmoid colon, rectum)	326 (59.8)	35 (44.9)	
Morphology	0-IIa	305 (56.0)	51 (65.4)	0.05
	0-Is	225 (41.3)	26 (33.3)	
	0-Ip	15 (2.7)	1 (1.3)	

Table 6 illustrates the reduction in low-confidence cases following the enhancement of criteria according to the NICE classification with the addition of the DF mode. Most low-confidence instances were attributed to challenges in evaluating vessel and surface criteria (71.8% and 53.8%, respectively). Remarkably, there was a significant improvement in both vessel and surface criteria (p<0.001), while no significant change was observed in the color criteria (p=0.15).

**Table 6:** Improvement of criteria after dual-focus magnification according to the NICE classification NBI: narrow banding imaging; NBI-DF: narrow banding imaging with dual focus; NICE: NBI International Colorectal Endoscopy p: McNemar test

Criteria	Low-confidence cases (N=78)		p-value
	NBI, n (%)	NBI+NBI-DF, n (%)	
Color	12 (15.4)	10 (12.8)	0.15
Vessel	56 (71.8)	32 (41.0)	<0.001
Surface	42 (53.8)	31 (39.7)	<0.001

## Discussion

In this study, we demonstrated that DF magnification significantly improved the rate of high-confidence NBI-based optical colorectal neoplasms in a Vietnamese real-time setting, especially for those with a length ≤ 5 mm. Additionally, the application of DF increased the accuracy and sensitivity of optical predictions, particularly for lesions initially categorized with low confidence when relying solely on NBI.

Previous studies, including our earlier study, have shown an improvement in optical diagnosis after using DF mode with high accuracy (90.2-96.6%) [5,6,10,13,14]. In terms of confidence levels, in NBI mode alone, lesions categorized with low confidence also exhibited significantly smaller sizes than those classified with high confidence (3.4 ± 2.1 vs. 3.9± 2.2, p=0.03). Consistent with earlier observational studies, within the subgroup of lesions measuring ≤5 mm in the current study, the introduction of NBI-DF led to a substantial increase in the rates of high confidence in predicting neoplastic lesions. Specifically, the proportions of high confidence level ratings in the NBI and NBI+NBI-DF groups were 87.1% and 93.1%, respectively (p<0.001) [5,6,13,14]. This finding underscores the consistent trend of enhanced confidence levels, particularly in the smaller lesion category, when utilizing NBI-DF compared to NBI alone.

Moreover, NBI+NBI-DF increased confidence in predicting initially low-confidence-classified polyps by NBI, with approximately 53.8% (n=42) of the 78 low-confidence patients shifting to high confidence. This resulted in a notable improvement in sensitivity and accuracy, increasing from 51.6% to 76.2% and 51.6% to 76.5%, respectively. Moreover, the addition of the DF mode significantly enhanced optical diagnosis in terms of surface and vessel criteria (p<0.001). Our findings suggest that DF magnification is particularly beneficial when achieving a high-confidence optical diagnosis is challenging without NBI alone, particularly in patients with a group size ≤ 5 mm.

Earlier research has documented the utility of magnifying NBI for evaluating the microvascular and surface structures of premalignant lesions, including characteristics like mesh capillaries and oval and tubular white structures [4-6,8]. Endoscopists can gain valuable insights into optical prediction with DF mode, which can be easily activated with a button press. The DF mode magnifies images using natural optical methods without sacrificing image resolution. It can provide magnifications of up to 100 times, with a field depth ranging from 2mm to 100 mm. In contrast to traditional magnification, which requires bringing the scope close to the lesion for a clear image (depth of field 1.5-3.0 mm), the DF mode allows convenient observation of an enlarged, close-up image by positioning the scope tip as close as 2 mm from the mucosa, with a relatively broad depth of field (3.0-7.0 mm) [9]. The DF mode assists endoscopists in achieving high magnification, revealing intricate details on the polyp surface, including vessel and surface patterns, without compromising resolution.

**Figure 3:**
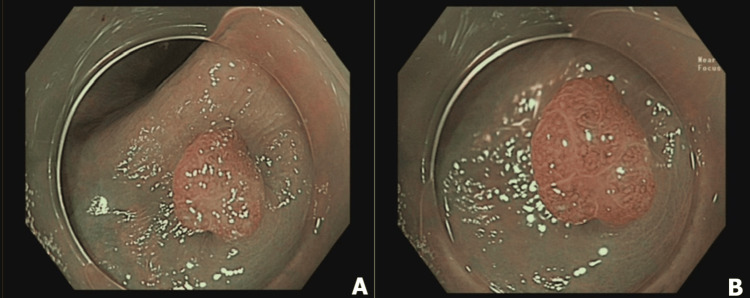
Polyp assessment in NBI and NBI+NBI-DF modes Polypoid lesion (0–Is) measuring 5 mm in size. A flat, light brown lesion initially classified as NICE 2 with a low-confidence prediction by NBI (A) and later confirmed as NICE 2 with a high-confidence prediction by NBI-DF (B). Dual focus mode facilitates the magnification of intricate microvascular and surface patterns. Histological examination confirmed a tubular adenoma with low-grade dysplasia. NBI: narrow banding imaging; NBI-DF: narrow banding imaging with dual focus; NICE: NBI International Colorectal Endoscopy

Consequently, the higher level of detail enhances the endoscopist's confidence in providing an optical diagnosis. Moreover, the improvement in confidence levels is due to the prefreezing function, which identifies the sharpest image from the preceding 4-29 frames. These factors result in fewer image capture attempts for high-quality photos and contribute to higher subjective image quality scores [15]. These capabilities could prove beneficial in real-time scenarios (Figure 3).

However, the percentage of high-confidence predictions for all polyps with NBI was consistent when DF was used. In our study, no notable advantage was observed for NBI+NBI-DF in the performance characteristics of optical diagnoses for lesions made with high confidence in NBI mode. Additionally, there was a decline in diagnostic performance for those who had low confidence. The 190 series might be confusing to interpret by offering optical clues to pathology that are not yet comprehended or providing additional details. Of non-neoplastic lesions, 3-6% may exhibit false-positive signs such as dark surfaces or visible blood vessels, like mesh capillaries, potentially leading to a misdiagnosis [2, 3]. These polyps may possess a thinner epithelial layer; others may exhibit congested surfaces due to inflammation. These factors can impact endoscopic prediction with NBI. Moreover, errors in optical diagnosis may be attributed to endoscopist-related factors, including poor photo documentation, failures of current classification systems, and incomplete histology [16]. Therefore, it is necessary to resect polyps diagnosed with low confidence in both modes (NBI and NBI-DF) and submit them for histopathological analysis.

The strengths of our study include its prospective design and the typical in vivo real-time setting for every endoscopic and optical diagnosis. A single endoscopist conducted all procedures and histologic predictions to mitigate potential investigator bias, mirroring real-world clinical practice. The endoscopist's awareness of polyp position, gross morphology, and size further contributed to comprehensive characterization, enhancing the clinical relevance of the study's findings. These aspects strengthen the implications of NBI and DF for endoscopic prediction of colorectal polyp histology.

Our study has certain limitations that warrant consideration. First, our findings may have limited applicability to community practice in Vietnam due to the underutilization of DF magnification. However, the increasing global availability of DF magnifying endoscopes indicates a potential trend in routine clinical practice. Second, we chose a non-randomized study design to assess the rate of high confidence in the NBI-DF mode following NBI application in daily clinical practice. A randomized design (NBI vs NBI-DF) would have deviated from our typical practice, initiating concerns about divergence. Third, our single-center study involving two experienced endoscopists and one pathologist provides valuable insights, but broader validation through multi-center studies is crucial for generalizability. The limited number of experienced endoscopists emphasizes the challenges for inexperienced practitioners, underscoring the need for standardized educational tools. Finally, our study could not make histologic predictions of neoplastic polyps or assess the diagnostic performance of endoscopy for distinguishing neoplastic polyps based on histologic grade.

## Conclusions

DF magnification holds promise for improving the rate of high confidence in the optical diagnosis of small colorectal polyps, particularly those ≤ 5mm, in the Vietnamese setting where optical magnification is limited. This technique also shows potential for enhancing diagnostic yield in routine clinical practice. Further multi-center research is warranted to assess its utility.
